# Case of a Wound in the Omentum

**Published:** 1803-10-01

**Authors:** J. Stables

**Affiliations:** Storsforth, Yorkshire


*Storsforth, Yorkshire,*

*Sept.* 11, 1803.

J. STABLES.

---

*Case of a* WOUND *in the* OMENTUM; *communicated by* J. STABLES, *of Storsforth, Yorkshire.*

J. G. a boy about twelve years old, received a stab from a sharp pointed knife, which penetrated the abdomen, about five inches obliquely above the right ileum. When I got to him, which was about an hour after the accident, I found a large portion of omentum protruding through the wound in his body, whose aperture might be about two inches in breadth. The lad had lost a good deal of blood when I got to him, from an artery in the omentum, which I secured as soon as possible, by needle and ligature, leaving a sufficient length of ligature, when the omentum was returned, to hang out below the orifice of the wound. Not finding the omentum much hurt, after washing from extraneous matter, &c. I returned it with all possible speed into the abdomen, and closed the wound with a suture and empl. adhesiv. &c. Gave him a large dose of opiate immediately, and the next morning opened his bowels with a solution of the sal. cathart. amar. For the first two days he went on very well; but on the third morning, I found him with strong febrile symptoms upon him, and complaining of a good deal of pain in his body. On examination, I found tension and inflammation had taken place over the lower part of the abdomen to a great extent. Having lost a good deal of blood from the wounded artery, and being a delicate boy, and considering the state of his pulse, I did not think it proper to deprive him of any more blood, but confined my treatment to the diligent use of emollient fomentations and poultices over the abdomen, and the frequent use of emollient enemas, &c. and opiates p. r. n. By persisting vigorously in the above antiphlogistic plan of treatment, the inflammation was unexpectedly subdued, and terminated in a state of suppuration. I opened the abscess and let out a large quantity of matter and coagulated blood, and also two two large portions of omentum came away along with it at different times; after which nothing unpleasant took place. By the use of common dressings to the wound and the free use of the cortex, and a generous diet, the boy, in the space of about a fortnight, got quite well.

